# Atypical sympathetic arousal in children with autism spectrum disorder and its association with anxiety symptomatology

**DOI:** 10.1186/s13229-015-0057-5

**Published:** 2015-12-11

**Authors:** Sakeena Panju, Jessica Brian, Annie Dupuis, Evdokia Anagnostou, Azadeh Kushki

**Affiliations:** Division of Engineering Science, University of Toronto, 35 St. George Street, M5S 1A4 Toronto, Canada; Bloorview Research Institute, 150 Kilgour Road, M4G 1R8 Toronto, Canada; The Hospital for Sick Children, 555 University Avenue, M5G 1X8 Toronto, Canada; The Institute of Biomaterials and Biomedical Engineering, University of Toronto, 164 College Street, M5S 3G9 Toronto, Canada

**Keywords:** Autistic disorder, Autonomic nervous system, Electrodermal activity, Galvanic skin response, Anxiety

## Abstract

**Background:**

Autism spectrum disorder (ASD) has been associated with autonomic atypicalities, although the nature of these differences remains largely unknown. Moreover, existing literature suggests large variability in autonomic function in ASD, motivating the need to examine the existence of subgroups that exhibit more homogeneous autonomic features.

**Methods:**

Electrodermal activity (EDA), a non-invasive physiological indicator of autonomic activity, was measured in typically developing children (*n* = 33) and those with ASD (*n* = 38) as participants performed tasks that elicit anxiety, attention, response inhibition, and social cognition processes. The ASD group was divided into low- (*n* = 18) and high-anxiety (*n* = 20) participants, and the groups were compared to mean EDA level and electrodermal reactions frequency (EDR).

**Results:**

The ASD group had a significantly blunted mean EDA response to the anxiety tasks (*p* < 0.004). The EDR response to all tasks, except response inhibition, was also blunted in the ASD group (*p <* 0*.*04). For this group, EDR frequency during the anxiety and social cognition tasks was negatively correlated with behavioral scores in the domains that were probed by each task (*p <* 0*.*002). The high-anxiety ASD group showed significantly decreased mean EDA compared to both the low-anxiety ASD group (*p* = 0*.*02) and the typically developing control group (*p* = 0*.*04). The high-anxiety ASD group also had significantly more severe symptoms than the low-anxiety ASD group on domains related to anxiety, attention, rule breaking, aggression, obsessions and compulsions, and depression.

**Conclusions:**

Our results suggest atypical autonomic function in children with ASD, specifically with respect to sympathetic activity. Moreover, anxiety symptomatology defined subgroups with distinct physiological and behavioral profiles. Overall, the results add to the body of literature supporting autonomic dysfunction in ASD and highlight the role of anxiety and autonomic features in explaining the variability in the autism spectrum.

## Background

There is growing evidence that autism spectrum disorder (ASD) is associated with dysregulation of the autonomic nervous system (ANS). The ANS is responsible for maintaining allostasis and regulating visceral functions through efferent and afferent connections to the central nervous system. The ANS is divided into three divisions namely, sympathetic, parasympathetic, and enteric branches. Sympathetic and parasympathetic outflows originate in the preganglionic neurons located in the spinal cord (intermediolateral cell column of the first thoracic to second lumbar segments) and the brainstem and sacral spinal regions (second to fourth sacral segments), respectively [1]. The sympathetic branch of the ANS is activated in response to stress to modulate the “fight” or “flight” response. Physiological changes accompanying this response include increased blood flow in the skeletal muscles, increased heart rate, blood pressure, and perspiration, and pupillary dilation. Activation of the parasympathetic branch is associated with the restoration and conservation of energy, with physiological effects generally opposite to that of the fight or flight response. The two branches of the ANS interact in a complex manner to maintain allostasis. Although existing evidence on ANS function in ASD is mixed [2, 3], an emerging body of literature suggests that ASD may be associated with hyper-arousal of the ANS. Evidence supporting this notion includes increased cardiac activity (elevated heart rate [4–8], decreased parasympathetic tone [5–7, 9], and larger tonic pupil size [10, 11]). In addition to these findings, altered ANS response to environmental challenges has also been reported in ASD [2, 3, 8]. Most notably, these findings include atypically blunted ANS activity in response to anxiety and psychosocial challenges [4, 12–15].

ANS hyper-arousal may be related to sympathetic hyper-arousal, parasympathetic undertone, or atypical interaction of the two systems. These possibilities can be tested using different measures of ANS function that quantify the individual effects of its two branches or their combined effects. Most ASD research to date has focused on either combined ANS function (e.g., heart rate, pupil size) or parasympathetic activity (e.g., heart rate variability). The latter has been of special interest due to its potential role in regulating emotional and behavioral functions [16]. The findings in this area are inconsistent and include both decreased and unaltered parasympathetic tone [2, 3]. Decreased parasympathetic tone, measured by respiratory sinus arrhythmia, has also been associated with social and emotional difficulties in ASD [9]. Given that the ANS output depends on the complex interplay of both its branches, it is critical to better understand sympathetic function in this population [3]. However, research on sympathetic function in ASD has been very limited.

To address the gap in characterizing sympathetic function in ASD, the first objective of the present study was to compare measures of electrodermal activity (EDA) between typically developing children and those with ASD. EDA is a non-invasive measure of the electrical conductance of the skin, which is affected by the activity of the eccrine sweat glands. These glands have predominantly sympathetic cholinergic innervation; therefore, skin conductance is suggested to provide a relatively “undiluted” measure of sympathetic activity [17]. Existing studies in this area are sparse and have reported mixed findings. These include both increased [18] and unaltered [19–22] basal skin conductance levels and decreased number of EDA reactions to affective images [23] and at rest [19], as well as atypical EDA reactivity to faces [20, 24], emotional judgment tasks [25], eye contact [26], and auditory stimuli [18]. Two studies [22, 27] studied EDA reactivity to anxiogenic stimuli and did not find significant differences between children with ASD and their typically developing peers in skin conductance levels, but an atypical pattern of electrodermal reaction frequency in the ASD group was reported [27].

The discrepancies in existing literature on ANS function in ASD suggest large heterogeneity in this population, though specific variables that can explain the variability in these physiological findings remain to be explored [2]. A recent study [15] suggested that anxiety symptomatology may explain some of this variability. This study showed that a sample of children with ASD and anxiety had atypically blunted cardiac and cortisol responses to psychosocial stress compared to children with only ASD and to typical controls. The second objective of this paper is to replicate the results of [15] in terms of the role of anxiety symptomatology in ANS function in ASD but with a specific focus on sympathetic activity measured by electrodermal activity. To our knowledge, this association has not been previously investigated.

## Methods

For this study, we used data from a sample of children with ASD (*n* = 47) and typically developing (TD) children (*n* = 37). The data from this sample were previously reported in [4] with respect to cardiac activity. Participants in the TD group did not have a diagnosis of ASD or any other developmental, neurological, or psychiatric disorders and were not born prematurely (*>*35-week gestational age). Participants in the ASD group had a primary clinical diagnosis of ASD supported by the Autism Diagnostic Observation Schedule (ADOS) [28] and the Autism Diagnostic Interview-Revised (ADI-R) [29]. All participants had a full-scale IQ greater than 50.

The Holland Bloorview research ethics board approved the study. Participants deemed to have capacity for consent, provided written consent. For all other participants, assent and written consent were obtained from the children and their legal guardians, respectively.

### Procedures

This experimental protocol is described in detail in [4] and summarized in Table 1. Participants completed five tasks eliciting performance anxiety (Stroop Word-Color Interference task [30]), social anxiety (public speaking task [12, 22]), attention (Rapid Visual Information Processing task [31, 32]), response inhibition (Stop Signal task [33]), and social cognition (Reading the Mind in the Eyes task [34, 35]). The Stroop task requires participants to name the color of words that spell out names of colors. For the public speaking task, participants were given 3 min to prepare a 3-min talk and deliver the talk to a panel of three people. Both the Stroop [27] and public speaking tasks [12, 15] have been used successfully in the literature as anxiogenic stimuli in this population. The Rapid Visual Information Processing task required participants to detect pre-defined sequences of three numbers in a series of randomly presented digits. For the Stop Signal Task, participants were asked to press the left and right buttons on a gamepad in response to X’s and O’s presented on a computer screen and to inhibit the response when an auditory tone was heard. Finally, participants viewed a set of 28 photos of human eyes and were asked to choose one of four words that best described what the pictured person was feeling or thinking. Each of the five tasks described above was preceded and followed by a baseline activity (movie-watching). For the baseline activity, participants watched clips from five animated movies (Toy Story, Lion King, Ice Age, Finding Nemo, and Happy Feet). Each task, except for public speaking, was also preceded by a practice period during which the participants were trained on the task and asked to demonstrate their comprehension of the task in a trial run.

**Table 1:** Outline of tasks performed during experimental session

Task	Target domain
Stroop Word Color Interference task	Anxiety
Public speaking	Anxiety
Rapid Visual Information Processing (CANTLAB)	Attention, working memory
Stop Signal	Response inhibition
Reading the Mind in the Eyes	Social cognition

### Measures

Intellectual functioning was assessed in both groups using the Wechsler Scales of Intelligence (Wechsler Abbreviated Scale of Intelligence (I and II) and the Wechsler Intelligence Scale for Children 4). For one participant in the TD group, an existing intelligence score from the Stanford–Binet Intelligence Scale was used. ASD symptom severity and anxiety was measured using the Social Communication Questionnaire. To assess emotional and behavioral characteristics of participants, the Child Behavior Checklist (CBCL/6-18) was used. Anxiety symptoms were assessed using the CBCL and the Revised Children's Anxiety and Depression Scale (RCADS).

EDA was measured using a wearable sensor from Shimmer Research. EDA was measured as skin conductance using a pair of 10-mm-diameter dry Ag-AgCl electrodes secured to the palmar surface of the proximal phalanges of the third and fourth digits of the non-dominant hand. Skin temperature was measured using a thermistor fastened to the palmar surface of the distal phalanx of the fifth digit of the hand. Hand movement was measured using an onboard triaxial accelerometer. The EDA time series was sampled at 256 Hz, transmitted over bluetooth to a laptop computer, and analyzed offline using MATLAB. To remove artifacts, EDA signals were filtered using a tenth order lowpass Butterworth filter with cutoff frequency of 1 Hz. The cutoff frequency was chosen in consideration of postganglionic sudomotor fiber firing rate of 0.62Hz [36] as well as existing literature [37, 38]. The signals were then detrended to eliminate linear increases in EDA due to improved adhesion of sensors over the course of the experiment. Signal peaks that did not show typical electrodermal response characteristics (rise time of 1–3 s, half-recovery time of 2–10 s, and an amplitude of 0.1–1.0 μs [18]) were identified as outliers and removed. Electrodermal responses (EDR) were identified as peaks in the signal with a minimum height of 0.05 μs [18] and inter-peak distance of 1 s. EDA values were log transformed to reduce data skewness [17]. Mean EDA level and frequency of EDRs were computed for each task and baseline interval. To ensure comparability of tasks and to minimize carryover effects, the analyses were performed using the first and last 3 min of each task and baseline interval, respectively.

Statistical analyses were performed using SAS version 9.4 (SAS Institute, Cary, NC). The effects of group and group *x* task interaction on EDA mean were examined using repeated measures multiple linear regression analysis. For EDR frequency, poisson regression with a log-linear model was employed to investigate the group and group *x* task interaction effects. To examine the effect of anxiety symptomatology on EDA, the ASD participants were divided into low- and high-anxiety groups based on their t-score on the CBCL anxiety problems subscale (low anxiety *<*65, high anxiety *≥* 65). The t-score of 65 was used as this is the cutoff for the borderline clinical range for the CBCL anxiety problems subscale. In all models, full-scale IQ and sex were included to account for group differences on these variables. Age, mean skin temperature, and medication status were also used as covariates as they are known to affect EDA signals [17, 27]. Contrast statements were conducted to examine group differences in task reactivity.

## Results

### Participants

The demographic information for the participants is shown in Table 2. Data for four participants in each group were excluded from analysis due to technical difficulties during data collection. Three participants from the ASD group were further excluded because they did not comply with the study protocol. Two more participants from this group were excluded due to excessive movement artifact and a fire alarm that disrupted the session. Compared to the TD group, the ASD group had significantly lower full-scale IQ (*p* < 0.0001) and significantly higher male to female proportion (*p* = 0.0176).

**Table 2:** Participant characteristics (typically developing and ASD groups)

	TD (*n* = 33)	ASD (*n* = 38)	Group difference
			(*p* value)
Age	12.5 *±* 2.9	12.1 *±* 2.9	n.s.
	(range 8–18)	(range 7–17)	
Full-scale IQ	113.1 *±* 14.1	94.3 *±* 20.2	<0.0001
	(range 85–140)	(range 53–146)	
Sex (male to female)	19:14	32:6	Fisher’s exact test
			0.0176
SCQ		18.1 ± 8.1	

*SCQ* Social Communication Questionnaire

*n.s* not significant

Of the 38 resulting participants in the ASD group, 8 were taking medications at the time of the study. These included serotonin norepinephrine reuptake inhibitors (SNRIs) (Strattrera), selective serotonin reuptake inhibitors (SSRIs) (Prozac, Zoloft, Citalopram), stimulants (Ritalin, Biphentin, Concerta), and atypical antipsychotics (Risperidone, Abilify).

The demographic information for the two ASD groups (low and high anxiety) is shown in Table 3. The groups did not differ significantly on age, IQ, or sex proportions. However, the high-anxiety group had significantly higher scores than the low-anxiety group on the Social Communication Questionnaire (SCQ), CBCL syndrome subscales of anxious/depressed, social problems, thought problems, attention problems, rule breaking, and aggressive behaviors, as well as all RCAD subscales.

**Table 3:** Participant characteristics (low- and high-anxiety groups)

	Low-anx. ASD (*n* = 18)	High-anx. ASD (*n* = 20)	Group difference (*p* value)
Demographics			
Age	11.7 (10.3, 13.1)	12.5 (11.2, 13.8)	0.4144
Full-scale IQ	98.0 (88.4, 107.7)	91.0 (81.8, 100.2)	0.2929
Sex (m:f)	15:3	17:3	0.7206
Medication (off:on)	14:4	16:4	0.7142
SCQ	14.7 (10.9, 18.6)	20.8 (17.4, 24.2)	0.0232
CBCL syndrome scales			
Anxious/depressed	55.6 (51.2, 60.0)	71.0 (66.9, 75.1)	<0.0001^a^
Withdrawn/depressed	56.0 (49.7, 62.3)	63.2 (57.4, 69.0)	0.0832
Somatic complaints	54.6 (50.9, 58.4)	60.8 (57.3, 64.3)	0.0207
Social problems	59.1 (54.5, 63.6)	67.6 (63.4, 71.7)	0.0063
Thought problems	59.9 (56.4, 63.4)	70.1 (66.9, 73.3)	0.0001^a^
Attention problems	60.8 (57.1, 64.5)	69.0 (65.6, 72.4)	0.0021^a^
Rule breaking	51.3 (47.4, 55.2)	60.7 (57.0, 64.3)	0.0011^a^
Aggressive behavior	54.7 (49.9, 59.5)	65.3 (60.9, 69.7)	0.0023^a^
RCADS			
Anxiety total	51.0 (44.1, 57.9)	65.1 (59.0, 71.1)	0.0040
Separation anxiety	54.2 (45.8, 62.6)	68.7 (61.4, 75.9)	0.0081
Generalized anxiety	52.2 (44.0, 60.4)	64.1 (57.0, 71.1)	0.0340
Panic disorder	50.6 (41.8, 59.4)	63.7 (56.0, 71.3)	0.0293
Social phobia	48.5 (43.0, 54.1)	57.5 (52.7, 62.3)	0.0176
Obsessive-compulsive	48.5 (43.5, 53.5)	58.9 (54.5,63.2)	0.0032
Depression	51.1 (44.2, 58.1)	65.0 (59.1, 71.1)	0.0021^a^

*CBCL* Child Behavior Checklist, *RCADS* Revised Children's Anxiety and Depression Scale, *SCQ* Social Communication Questionnaire

^a^Significant at *α* = 0.0025 level after family-wise Bonferroni correction (20 tests)

### Movements

Total acceleration, computed as the norm of the acceleration vector, did not differ significantly between the ASD and TD groups.

### EDA measures

**Fig. 1:**
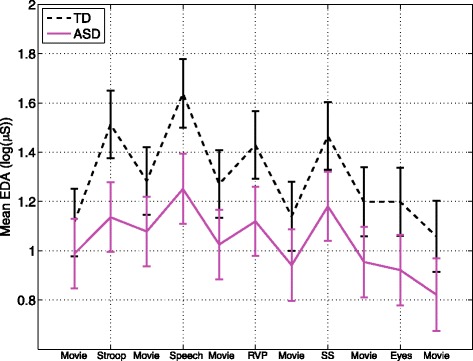
Mean EDA across tasks for the TD and ASD groups. *Error bars* represent standard error. Reactivity to Stroop and public speaking tasks (task—baseline) was significantly blunted in the ASD group (*p* < .0004)

Repeated measures analysis revealed a significant group *x* task interaction effect on mean EDA (*F*(10,690) = 3.14, *p* = 0*.*0006; Fig. 1). Post hoc analysis showed blunted reactivity (task-preceding baseline) to the Stroop (estimated group difference = 0.25 *±* 0.06, *t*(690) = 4.44, *p <* 0*.*0001) and public speaking tasks (estimated group difference = 0.18 *±* 0.06, *t*(690) = 2.94, *p* = 0*.*0034) in the ASD group. For both groups, mean EDA increased significantly from the preceding baseline in response to all tasks (*p* < 0.0001), except for the Reading the Mind in the Eyes task. For the ASD group, the increase in response to the Rapid Visual Information Processing was also insignificant after correction for multiple comparisons (*p* = 0.0129).

**Fig. 2:**
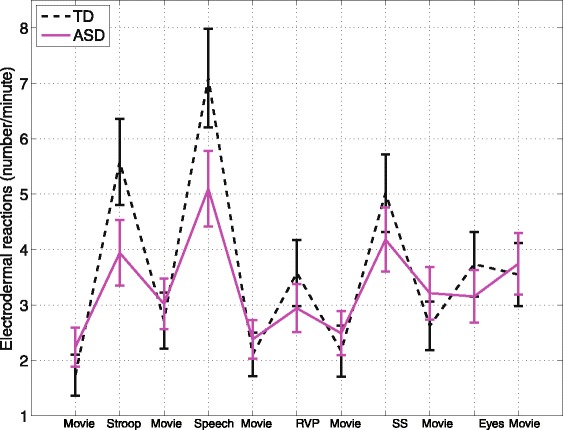
EDR frequency across tasks for the TD and ASD groups. *Error bars* represent standard error. ASD group had blunted reactivity (task—baseline) to all tasks (*p* < 0.04), except for the Stop Signal task where the difference was marginally significant (*p* = 0.06)

EDR frequencies for the TD and ASD groups are shown in Fig. 2. There was a marginally significant group *x* task interaction on the EDR frequency (*χ*^2^(10) = 18.5, *p* = 0.0469). Post hoc comparisons showed that the ASD group had blunted reactivity (task-preceding baseline) to all tasks (Stroop *p = 0.*0017, public speaking *p* = 0.0112, Rapid Visual Information Processing *p* = 0.0376, Reading the Mind in the Eyes *p* = 0.0086), except for the Stop Signal task where the difference was marginally significant (*p* = 0*.*0632). For the TD group, EDR frequency increased significantly from the preceding baseline in response to all tasks (*p* < .0001). For the ASD group, EDR frequency increased significantly from the preceding baseline to all tasks (*p* < 0.0001), except for the Rapid Visual Information Processing and the Reading the Mind in the Eyes tasks.

Within the ASD group, EDR frequency during the anxiety and social cognition tasks was negatively correlated with the behavioral scores in the domains that were probed by each task (Stroop/CBCL anxiety problems: regression coefficient estimate = −0.03, *p* = 0*.*0011; public speaking/CBCL anxiety problems: regression coefficient estimate = −0.02, *p* = 0*.*0249; Reading the Mind in the Eyes task/CBCL Social: regression coefficient estimate = −0.03, *p* = 0*.*0046).

### Anxiety group

**Fig. 3:**
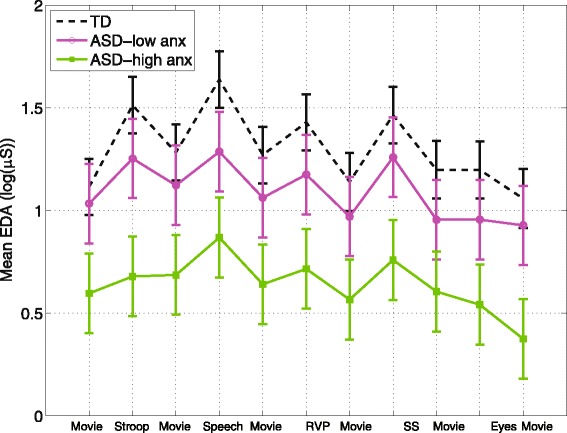
Mean EDA across tasks for the high- and low-anxiety groups. *Error bars* represent standard error. The high-anxiety ASD group had significantly decreased mean EDA compared to the ASD-low anxiety and control groups (*p* = 0.04)

Mean EDA for the TD, low-anxiety ASD, and high-anxiety ASD groups is shown in Fig. 3. There was a significant effect of anxiety group (low versus high) on mean EDA, with the high-anxiety group showing significantly decreased mean EDA overall (estimated group difference = 0.46 *±* 0.20, *t*(364) = 2.35, *p* = 0*.*0193). The anxiety group *x* task interaction was not significant. Comparing the anxiety groups to the typical controls revealed a significant difference between the controls and the high-anxiety group overall (estimated group difference = 0.50 *±* 0.24, *t*(700) = 2.02, *p* = 0*.*0436), but not between the TD and the low-anxiety group. There was no significant correlation between the EDA measures for the anxiety groups and scores on the SCQ, CBCL, and RCADS.

**Fig. 4:**
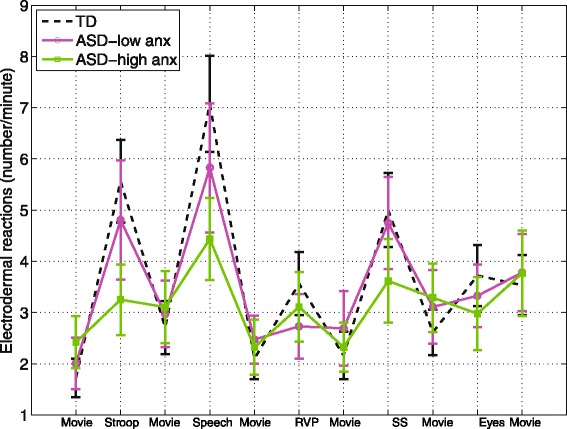
EDR frequency across tasks for the high- and low-anxiety groups. *Error bars* represent standard error. EDR frequency was marginally higher in the low-anxiety group (*p* = 0.06)

EDR frequency was marginally higher in the low-anxiety group (Fig. 4; low anxiety 3.77 *±* 0.69, high anxiety 2.92 *±* 0.54, *z* = 1.85, *p* = 0*.*0646), but the anxiety group *x* task interaction term was not significant. EDR frequency was not significantly different between the controls and either anxiety group.

As exploratory analysis, we examined the effect of group membership on the EDA measures when the groups were created based on scores on other CBCL comorbidity domains (somatic, attention, rule breaking, aggression). No significant effects were found after correcting for multiple comparisons.

## Discussion

The main findings in the current study were (1) atypical EDA in the ASD group characterized by a blunted mean EDA reactivity to the anxiety tasks and decreased EDR reactivity in all tasks, (2) negative correlation between EDR frequency and behavioral scores in the anxiety and social domains in the ASD group, and (3) differential mean EDA and behavioral patterns between the low- and high-anxiety subgroups within the ASD group.

### Atypical electrodermal activity in ASD

Given the findings in previous literature, we expected that the ASD group would exhibit sympathetic hyperarousal evidenced by atypically increased or decreased EDA level and increased EDR frequency. Although a trend toward decreased mean EDA was observed in the ASD group overall, group differences did not reach statistical significance for any of the EDA measures in our study collapsed across conditions. This may be related to the large variability in autonomic function in this sample. As discussed in the next section, anxiety symptomatology may explain some of this variance and could be used to derive more homogeneous subgroups with respect to electrodermal activity.

Our data also showed that ASD was associated with blunted reactivity to tasks eliciting anxiety, attention, response inhibition (marginally significant), and social cognition. Blunted reactivity to psychosocial challenges in ASD has been previously reported in the literature both in the context of autonomic cardiac measures [4, 12, 15] and hypothalamic–pituitary–adrenal axis function [15]. The present study adds to this literature by revealing a dampened response to other mental and cognitive tasks. Such dampened reactivity has also been reported across a number of psychiatric and affective conditions including attention deficit/hyperactivity and conduct disorder [39], alexithymia [40], depression [41], and high trait anxiety [42, 43]. The inability to regulate physiological responses to environmental stimuli may also be related to emotion regulation difficulties. These difficulties, together with impaired attentional control, may play a role in the early emergence of ASD symptoms [44] (e.g., by hindering the experience of positive associations from interactions with others). Interestingly, markers of a well-regulated ANS have been associated with improved social function in children with ASD [45]. Further research is needed to understand the associations between ANS atypicalities and ASD symptomatology.

It is important to note that the literature findings on mean EDA reactivity to anxiogenic stimuli are mixed. Specifically, two previous studies did not find atypical mean EDA reactivity to anxiogenic stimuli in ASD during the Stroop [27] and phychosocial challenges [22]. The discrepancies may be related to differences in experimental conditions (e.g., nature of baseline activity) or sample characteristics (e.g., age, IQ, diagnosis status (ASD versus high-functioning autism), presence/exclusion of comorbid conditions) or may indicate a need for larger sampler sizes to capture significant differences in mean EDA, which exhibits high variability in this population.

Increased EDR frequency is generally associated with increased sympathetic activity. Our results therefore suggest decreased sympathetic reactivity in the ASD group. This may be indicative of a deficit in sympathetic modulation to meet task demands and associated with central/peripheral neurobiological differences in ASD. While there is currently no evidence to support differences in peripheral conduction, several neuroimaging/EDA studies suggest central influences on sympathetic atypicalities in ASD. In particular, a widespread network of regions, including the amygdalae, and the prefrontal, anterior cingulate, and insular cortices, has been associated with sympathetic modulation [46]. ASD has been associated with differences in neuroanatomy, function, and connectivity in these regions [47–49], which may affect sympathetic function. These regions have also been implicated in studies of social deficits in ASD [47] as well as in neuro-circuitry of anxiety [50]. It is therefore interesting that our results show a significant correlation between behavioral scores in these domains and decreased EDR frequency in the respective tasks. These results are also consistent with those of [15] which suggested a negative correlation between anxiety symptom severity and heart rate responsiveness to social stress.

Further supporting atypical central autonomic processing, a study of resting state activity in ASD [19] found that the EDR signal was positively correlated with activity in several regions involved in autonomic processing (e.g., anterior insular and cingulate cortices) in neurotypical controls, but not in the ASD group. In addition, the results of that paper also suggest that weaker default mode network connectivity in ASD may be partially explained by differences in EDA activity. Future neuroimaging studies are needed to further examine the relation among brain function, autonomic differences, and behavior in these domains.

The blunted task reactivity observed herein may also be related to compensatory down-regulation resulting from chronic exposure to stress [15]. This would be consistent with the high prevalence of comorbid anxiety in ASD [51] and previous reports of hyper-arousal in this population [5–8, 27]. In this context, reduced reactivity may reflect inhibitory coping effects [42].

Finally, other mechanisms may have contributed to decreased arousal during the tasks used in the study. These include deficits in allocation of attentional resources [39] or other executive functions as well as lower levels of motivation to performance, engagement, or interest in study tasks [40]. Future studies are needed to further investigate these issues.

### ASD subgroups

Our results show that when split based on anxiety symptomatology, two different subgroups emerge within the ASD group, with the high-anxiety group exhibiting significantly decreased mean EDA relative to both the low-anxiety ASD and TD groups. Our results also mirror those reported in [15] in which a high-anxiety group within ASD had lower heart rate than a low-anxiety ASD group. Overall, these results indicate that anxiety symptomatology may explain some of the variability in EDA findings in existing literature.

Paradoxically, decreased EDA may suggest both hyper- and hypo-arousal as the level of arousal input and physiological output are thought to follow an inverted U-shaped relation. In particular, arousal increases physiological output to a certain point, beyond which the physiological response decreases (a concept similar to Pavlov’s notion of transmarginal inhibition) [52]. Consistent with this model, individuals with high trait anxiety have been shown to exhibit diminished EDA levels [52]. Given that our groups were derived based on a measure of trait anxiety, our finding of decreased EDA levels may reflect hyper-arousal in this sample.

The pattern of decreased EDA was not task-specific in our data and was evident even during baseline phases. This may suggest that the observed EDA differences were likely not related to differences in responding to any particular task but to a more global physiological dysfunction or to differences in the overall experience of the experimental setting (e.g., coping with new environment and staff). Decreased levels of EDA have previously been reported in other populations with psychiatric difficulties including depression [17], anti-social behavior [53], and externalizing behavior disorders [54].

In addition to having distinct physiological profiles, the high- and low-anxiety ASD groups differed significantly on a number of behavioral domains. In particular, the high-anxiety group showed more severe symptomatology on measures of affective and psychological difficulties (CBCL anxious/depressed, thought problems, attention problems, rule breaking, and aggressive behavior; RCADS anxiety total (marginally significant), obsessive-compulsive (marginally significant), and depression subscales). These results suggest a different behavioral profile between the two anxiety groups. Our results complement those reported in [54] where adults with ASD who exhibited low EDA also showed poorer emotion recognition compared to those with higher EDA levels. Given the high rates of comorbidity in the high anxiety group, it remains possible that the findings are driven by the presence of greater comorbid symptoms overall (versus anxiety alone). Future research is needed to further understand the relation between anxiety and other comorbidities in this population.

Overall, our results suggest an interaction between sympathetic function and anxiety and ASD symptomatology. Future research is needed to further clarify the nature of these associations.

### Limitations

Some limitations of the present study are noteworthy. First, in this study, sympathetic function as measured by electrodermal activity was examined in isolation. The output of the autonomic nervous system, however, is a result of complex interactions among central and peripheral mechanisms that include both the sympathetic and parasympathetic systems as well as the neuroendocrine system. Further studies are needed to examine these systems simultaneously and to pinpoint system-level differences in this area.

The second limitation of this study was that the specific measures of EDA such as the orienting response and habituation were not examined. This was mainly due to the continuous nature of tasks. Future studies designed specifically for examining these measures (e.g., using discrete stimuli) can further shed light on the nature of EDA atypicalities in this population.

Our sample included participants who were receiving psychopharmacological interventions which may affect autonomic function. While we controlled for the effect of medications on EDA measures in our analyses, future studies with larger sample sizes are needed to quantify these effects.

Finally, given the large variability in EDA measures in this population, our sample size may have contributed to null findings on both the physiological and behavioral domains.

## Conclusions

In the present study, we examined sympathetic function in ASD as measured by electrodermal activity. Our main findings include (1) blunted electrodermal reactivity to tasks eliciting anxiety, sustained attention, and social cognition in ASD and (2) identification of two subgroups within the ASD sample based on anxiety symptomatology. The subgroups exhibited distinct physiological and behavioral profiles characterized by low EDA level and more severe symptoms in ASD, anxiety, attention, and behavioral domains in the high-anxiety group. Overall, the results add to the body of literature supporting autonomic dysfunction in ASD and highlight the role of anxiety and autonomic features in explaining the variability in the autism spectrum.

## Abbreviations

ANS: autonomic nervous system
ASD: autism spectrum disorder
CBCL: Child Behavior Checklist
EDA: electrodermal activity
EDR: electrodermal reaction
RCADS: Revised Children's Anxiety and Depression Scale
SCQ: Social Communication Questionnaire
TD: typically developing
